# Challenges in preparing and implementing a clinical trial at field level in an Ebola emergency: A case study in Guinea, West Africa

**DOI:** 10.1371/journal.pntd.0005545

**Published:** 2017-06-22

**Authors:** Sara Carazo Perez, Elin Folkesson, Xavier Anglaret, Abdoul-Habib Beavogui, Emmanuel Berbain, Alseny-Modet Camara, Evelyn Depoortere, Annabelle Lefevre, Piet Maes, Kristian Nødtvedt Malme, Jean-Marie Denis Malvy, Sien Ombelet, Geertrui Poelaert, Daouda Sissoko, Alexis Tounkara, Pierre Trbovic, Pascal Piguet, Annick Antierens

**Affiliations:** 1 Médecins Sans Frontières, Brussels, Belgium; 2 Department of Preventive and Social Medicine, Laval University, Québec City, Canada; 3 Inserm, UMR 1219, Université de Bordeaux, Bordeaux, France; 4 Programme PACCI, Abidjan, Côte d’Ivoire; 5 Centre de Formation et de Recherche en Santé Rurale de Mafèrinyah, Conackry, Guinea; 6 Médecins Sans Frontières, Conackry, Guinea; 7 Institute of Tropical Medicine, Antwerpen, Belgium; 8 European Mobile Laboratory Project, Hamburg, Germany; 9 KU Leuven, Rega Institute for Medical Research, Leuven, Belgium; 10 Pellegrin Hospital, Bordeaux, France; University of Geneva Hospitals, SWITZERLAND

## Abstract

During the large Ebola outbreak that affected West Africa in 2014 and 2015, studies were launched to evaluate potential treatments for the disease. A clinical trial to evaluate the effectiveness of the antiviral drug favipiravir was conducted in Guinea. This paper describes the main challenges of the implementation of the trial in the Ebola treatment center of Guéckédou. Following the principles of the Good Clinical Research Practices, we explored the aspects of the community’s communication and engagement, ethical conduct, trial protocol compliance, informed consent of participants, ongoing benefit/risk assessment, record keeping, confidentiality of patients and study data, and roles and responsibilities of the actors involved. We concluded that several challenges have to be addressed to successfully implement a clinical trial during an international medical emergency but that the potential for collaboration between research teams and humanitarian organizations needs to be highlighted.

## Introduction

The largest and most complex Ebola virus outbreak in history started in Guinea in December 2013 and spread rapidly to neighboring countries in March 2014 [1]. As no effective treatment existed against Ebola virus disease (EVD), Ebola treatment centers (ETC) were only able to provide supportive care with case-fatality rates above 50% [2].

In August 2014, the World Health Organization (WHO) convened an advisory panel that unanimously concluded it would be acceptable to assess potential unregistered interventions that had shown promising results in preclinical laboratory and animal phases but had not been fully evaluated in phase I or II trials in humans [3]. Médecins Sans Frontières (MSF), which was extensively involved in frontline care for Ebola patients, the French National Research Institute for Health and Medical Research (INSERM), and Guinean authorities created a partnership to conduct a “proof of concept” study on the efficacy of high-dose favipiravir in reducing mortality in patients with EVD. Favipiravir, a novel viral RNA polymerase inhibitor developed by the Japanese company Toyama/Fujifilm, was a prioritized drug on the WHO expert panel’s list because of its good human safety data and promising results against the Ebola virus in preclinical studies [4–8]. The multisite JIKI study, named after “hope” in the Malinke language, was a noncomparative, single-arm, open-label clinical trial [9]. Any patient aged 1 year or older with laboratory-confirmed EVD was offered the opportunity to participate in the trial and was asked for informed consent. The European Mobile Laboratory consortium (EMLAB) performed routine laboratory tests (Ebola virus PCR and biochemistry). The protocol of the trial has been described elsewhere [9].

With the recruitment of the first patient in the Guéckédou ETC on December 17, 2014, the JIKI trial became the first phase II study on EVD ever implemented by a research team in collaboration with an international nongovernmental organization. This unique situation was complicated by the international humanitarian crisis associated with the uncontrolled Ebola virus epidemic and a vulnerable resource-poor setting of rural Guinea.

This paper is a systematic assessment of the JIKI trial implementation in the field at the Guéckédou ETC, the first center involved in the study. It does not address the prefield aspects (methodological choices, writing of the protocol). As all authors of the present paper were involved in the trial, the assessment may be subjective. Our goal is to offer the scientific and clinical research communities critical feedback into the issues and challenges of this unique situation in order to highlight potential problems in future EVD outbreaks.

## Issues addressed

We will discuss the issues surrounding the implementation of the JIKI trial in the field in order to address the interaction between Good Clinical Research Practices [10] and the significant practical challenges in this particular context (Table 1).

**Table 1:** Key recommendations for a trial implementation in an Ebola outbreak.

Principle	Recommendation
Communication with community	Pretrial recruitment and engagement of community leaders in the trial planning and launch. Thoughtful preparation and pretest of messages about the trial.
Ethical conduct	Evaluation of the inclusion criteria based on equity and risk/benefit assessment.
Protocol compliance	Consensus among clinicians to adapt previous protocols to standard operational procedures (SOPs). Ongoing evaluation of acceptability of changes in patient management to accomplish with trial protocol.
Informed consent	Pretrial assessment of cultural adaptation of informed consent. Continuous training of specific staff to obtain informed consent. Local consultation for handling exceptional cases (e.g., unaccompanied children).
Ongoing benefit/risk assessment	Interpretation of laboratory results considering Ebola virus disease (EVD) infectivity. Daily analysis of mortality data.
Records	Innovative management of data recording in the Ebola virus high-risk setting: installation of a scanner, pictures of files, devices allowing decontamination.
Confidentiality	Communication of results as soon as possible to avoid rumors. Clear and early identification of communication channels with media and political leaders.
Roles and responsibilities	Clear definition of responsibilities and communication channels between all actors involved in the implementation (the French National Research Institute for Health and Medical Research [INSERM], Médecins Sans Frontières [MSF], Guinean authorities, the European Mobile Laboratory consortium [EMLAB]).

### Communication with the community

Communities from Forested Guinea were known to mistrust government and Western humanitarian aid agencies, which led to serious security incidents [11]. EVD was still unknown and many rumors were circulating such as that the disease did not exist; that it had been introduced purposely to reduce the population; and that ETC patients were experimented on, killed, or had their organs harvested [12, 13]. Rumors had to be countered, and the messages to the population on the trial objectives and implementation needed to be comprehensive and adapted to the context to minimize fear and rejection as well as inappropriate expectations. As the spread of the epidemic was unpredictable and a large region potentially affected, numerous villages had to be reached in a very short time.

To ensure the community involvement in the trial, local leaders, including women’s organizations, youth groups, religious leaders, and village elders, were invited by the research partners to participate in a Community Advisory Board (CAB). This was headed by the regional coordinator of the fight against Ebola and specifically created to counsel and support the trial implementation. During the preparation phase, the CAB discussed the trial protocol and the communication strategy for the community. Following their recommendations, the focus for communication was put on providing correct and comprehensive information to patients, their families, and all 350 MSF staff members, most of whom originated from Guéckédou prefecture, rather than informing entire communities. Anthropologists and key national staff collaborated to develop the form and content of the messages, which was tested on various members of the staff to ensure correct translation into local languages, sensitivity to local culture, and ease of understanding.

At the same time, a mobile health promotion team addressed rumors in the communities, countered misconceptions, and answered questions on the trial. They also relayed community responses to the trial team to modify the messages to patients and their families. For example, when a rumor arose that 4 EVD survivors treated with favipiravir became “crazy” after returning to their villages, a combined MSF and INSERM team visited the villages and discussed the issue with the families and the local health authorities.

However, the interventions were limited by the rapid geographical expansion of the epidemic. It was not possible to ensure that all villages in the ETC catchment area were included in the communications. Patients referred from prefectures outside Guéckédou would not have heard any information about the trial.

### Ethical conduct

Research with human subjects must ensure that 3 basic ethical principles are followed: respect for persons, beneficence, and justice [10, 14, 15]. Favipiravir was already shown to be a promising drug with the potential to improve EVD prognosis; it was therefore considered acceptable to evaluate this drug in EVD patients. Given the high mortality in children under 5 years [16, 17] and the fact that Ebola virus often strikes in clusters, including relatives [18], excluding children would certainly have compromised trial acceptability. Dose-finding or tolerance data in children were not available. A team of paediatric experts concluded that full maturation of the enzymes involved in the metabolism of favipiravir was achieved by 12 months. Weight-based doses were chosen based on adult dosages, for which safety data were available [19]. Children under the age of 1 year or weighing less than 10 kg had to be excluded; however, in practice, this resulted in the exclusion of only 2 infants.

Pregnant women were another particularly vulnerable group. Since no human studies of favipiravir in pregnancy had ever been performed and embryonic teratogenicity was reported in mice, no insurance company would cover the risk for pregnancy; thus, pregnant women had to be excluded from the JIKI trial. Pregnant women, however, had a higher case-fatality rate compared to nonpregnant women. The vast majority of pregnancies in EVD patients resulted in miscarriage or intrauterine death, and the few babies born died in a few days [20, 21].

To avoid denying pregnant women access to a potentially beneficial investigational product, MSF accepted responsibility and liability for the emergency use of favipiravir in this group. A protocol for monitored emergency use, including informed consent and rigorous data collection, was submitted for approval to the appropriate ethics committees. Pregnancy testing was introduced for all women of childbearing age admitted in the ETC.

Nonetheless, a time complication occurred, as the emergency-use protocol for pregnant women was still under ethics review when the trial started. This did not, however, have any practical impact, because no pregnant women were admitted in the ETC during the trial period.

### Protocol compliance

The trial’s standard operational procedures (SOPs) had to be incorporated into the MSF Ebola virus case management protocol without creating any negative impact on patient care or adding extra risks for the staff. To ensure compatibility with the routine management protocols already in place, the research team wrote the SOPs on site with MSF medical staff. Feasibility and organizational changes were evaluated before implementation to ensure smooth integration of the trial into clinical activities.

After trial initiation, the first challenge was related to the study endpoint, which was survival to day 14. Some patients met MSF’s discharge criteria (negative PCR results after 3 days without symptoms) before day 14 but still needed monitoring. Thus, a rehabilitation zone was built so that these patients could move more freely but still remain under surveillance of clinical staff.

The second challenge was the follow-up tests for the trial participants. Routine clinical procedures called for PCR testing at admission and at the end of symptoms, with no blood collection between these 2 samples. However, the trial protocol demanded extra sampling at day 2 and day 4, which increased risks for patients and staff. In practice, the additional venous sampling was considered acceptable as it included follow-up biochemical tests, which were beneficial to patient care. Patients were informed of the increased number of blood draws at the time of informed consent. The volume of each blood collection was limited to a minimum. The diagnostic laboratory adapted its organization to the new requirements, including new schedules and the incorporation of biochemical testing. The clinical staff was trained to use the biochemistry results in EVD practice. There were, however, some limitations related to these new procedures. Adding new tests at the laboratory led to some unexpected difficulties. The platform, Abaxis Piccolo Xpress analyzer, was sensitive to heat, and the laboratory did not have a cooling system. Disinfection of the machine was also a challenge, as the chlorine solution normally used harmed the electronic components.

The third challenge was the need for early initiation of treatment. As the antiviral medication was expected to be more effective the earlier in the course of the disease it was started, it was crucial to include patients in the trial as soon as possible. Treatment initiation, however, could only be done after the laboratory confirmed EVD and informed consent was obtained. This required important changes in the staff shifts to ensure that inclusion could be done as late as 1:00 AM. Moreover, the samples for PCR testing had to reach the diagnostic lab before 6:00 PM, due to the long process of decontaminating samples and performing the necessary tests. Consequently, some patients arriving late in the evening, mostly those transferred from other prefectures, had to wait almost 24 hours before inclusion.

### Informed consent

Closely related to the principle of respect for the dignity of participants, an informed consent had to be obtained from all patients before their inclusion in the trial [10]. During the JIKI trial, communication with potential participants was complicated by a setting of fear, mistrust, and the disease itself, as well as by clinical trial illiteracy. As consent was only requested from laboratory-confirmed Ebola virus patients, it had to be obtained in the ETC high-risk zone from medical staff wearing full personal protective equipment, which clearly hampered communication and the perception of free choice for the patient.

Some patients could not decide for themselves without extensive family consultation. In addition, there were no clear legal or traditional rules for ethically obtaining proxy consent for the inclusion of children or impaired persons who arrived at the ETC without guardians.

Several actions were taken to try to ensure a freely given and culturally adapted informed consent. Firstly, a multidisciplinary team of medical, health-promotion, psychosocial, anthropological, and research personnel translated the informed consent into comprehensive and culturally sensitive local languages. Secondly, oral consent was preferred over signed consent because of cultural acceptability; according to the local psychosocial team, signing a document while hospitalized was considered as signing a legal will. Therefore, informed oral consent was signed off by 2 witnesses or by the guardian in the case of minors.

Finally, the Guéckédou CAB was consulted regarding unaccompanied children and advised to seek special authorization from national authorities. The national coordinator of the fight against Ebola was contacted and accepted the responsibility for the inclusion of unaccompanied minor children in the trial.

However, despite efforts to explain the protocol in local languages, it was difficult to assess the amount of information understood by each patient, due to language and cultural barriers and the clinical status of patients.

### Ongoing benefit/risk assessment

In order to assure that the benefits continuously outweighed the potential risks associated with the investigational drug, strict pharmacovigilance of favipiravir was crucial, as no safety data were available for the high doses used in the trial. However, due to the severity of EVD and limited availability of clinical facilities, distinguishing potential adverse effects of the favipiravir treatment from the signs and symptoms of EVD was challenging.

During and after administration of favipiravir, the patients were closely monitored for any adverse signs or symptoms not corresponding to typical EVD symptoms, with a focus on those adverse events previously described in clinical studies with favipiravir for the treatment of influenza infection. Biochemical tests were used to observe possible liver and kidney damage. However, at the individual patient level, the interpretation of the causes of deterioration was still difficult, because some signs and symptoms, such as diarrhea, vomiting, or dizziness, could be related to either EVD or favipiravir.

Additionally, mortality data were sent to the Data Safety Monitoring Board (DSMB) on a daily basis. The protocol included rules that allowed for stopping the trial if interim analysis, planned after every 20 patients, showed that on-trial mortality did not trend lower than pretrial mortality.

### Records

The quality of the data collection is related to clinical staff qualifications as well as quality of the recording system. Therefore, medical personnel were trained on specific data-collection methods. What proved to be more challenging was handling these data. Due to the infection-control restrictions in the ETC, paper-recorded patient data could not leave the high-risk zone. To address this problem, case report forms and routine patient documents were scanned inside the high-risk zone, and the data were sent electronically to both the medical and research databases. Compared to the pretrial practice of verbal transmission of patient data between the high- and low-risk zones, this new procedure resulted in fewer transcription mistakes and less time for the staff in the high-risk zone.

### Confidentiality

The context of the trial, with high social, media, and political pressure, threatened the confidentiality of the study data. The trial was an open-label, single-arm study. After the trial was launched, most EVD-confirmed patients admitted into the ETC received favipiravir treatment. This was known by the staff and easy to grasp for the local communities. As the primary study outcome was mortality, confidentiality in regards to individual patient outcome and the potential efficacy of the favipiravir treatment was difficult to keep undisclosed to the outside world. In an attempt to limit the spread of rumors in the local community, regular meetings with the staff were held to explain the need for prudent interpretation of the observed outcomes and the importance of confidentiality regarding patients and trial results. The ETC teams and CAB were the first to be informed of interim and final results.

In February 2014, the trial DSMB and Scientific Advisory Board recommended that the results of the first interim analysis should be shared with the international scientific community. These results suggested good tolerance of the drug and the importance of baseline viral load as a key stratification factor in further final analysis, but they did not demonstrate the effectiveness of the drug. However, the research team could not control the interpretation of these interim results by the media and political leaders. Rumors circulated in the national and international media about favipiravir efficacy. With decreasing EVD incidence rates in Forested Guinea, it became obvious that the initial estimation of the patient cohort size would not be met. National authorities strongly urged the trial leaders to publicly announce the results before the trial completion in order to make the drug available in other Guinean ETCs.

### Roles and responsibilities

Both MSF and INSERM had little experience in the unique collaboration between a humanitarian organization and a typical life-science research institute.

The trial agreement stipulated that INSERM was responsible for compliance with the trial protocol and the quality of data collection and analysis, while MSF was responsible for patient management. Communication channels were improved when MSF appointed a research coordinator to coordinate trial preparation and patient care and to ensure full integration of the research and clinical teams.

Additional trial staff, financed by INSERM, were recruited with the same working contracts as MSF national staff and trained together with the other ETC staff in the trial SOPs. This process created a strong team spirit and a feeling of ownership of the project and avoided dividing the staff into 2 groups with different management and working conditions.

Field logistics were provided by MSF, while INSERM was responsible for official communications about the trial with the media and the authorities.

## Discussion

Implementing a phase II clinical trial during a devastating epidemic in a research-illiterate environment was full of challenges. Success required cultural sensitivity to build trust within communities and among patients, inclusion criteria based on equity and a risk/benefit assessment, close collaboration between research and clinical teams, and flexibility to adapt trial procedures to field constraints. The main pitfalls were related to protocol compliance: the technical lab problems after the introduction of new tests and the contradiction between study endpoint and clinical discharge criteria. Confidentiality of trial data was not fully controlled and required continuous surveillance.

In a context of fear and mistrust of international actors, community engagement was essential to ensure the appropriate implementation and progression of the trial. A pretrial initiative to inform and involve community leaders; the development of thoughtful, culturally appropriate messages; and a consensual community strategy were essential aspects of this engagement that should not be neglected in any Ebola virus trial.

Scientists have argued for [22–25] and against [26–28] the use of randomized controlled trials (RCTs) to evaluate Ebola therapies. Our paper focused on field implementation, but this was also influenced by the methodological choice of a single-arm design. With patients facing a high probability of mortality and despite the absence of previous efficacy studies, we believe that individual interests must prevail over purity of trial methodology. It would have been unethical and impractical to run an RCT demanding patients to consent to conventional, ineffective care when a potentially beneficial treatment was available [26, 27]. It is noteworthy that virtually all Ebola virus therapy clinical trials have had a single-arm design [28–31]. During the Ebola outbreak, compassionate use of unapproved treatments was criticized because it did not systematically contribute to building knowledge about therapeutic efficacy [23, 27]. In the JIKI trial, the use of favipiravir in pregnant women followed a WHO-recommended protocol for monitored emergency use [32].

In conclusion, the JIKI trial illustrated how an international research team collaborated both with a humanitarian medical organization and local researchers to improve scientific knowledge, create innovative partnerships, promote local capacity building in research, and help EVD patients. Our observations are context specific but may be useful to understand the complexity and challenges of establishing research activities during future international medical emergencies.

## Supporting information

S1 Text Trial protocol. (PDF)

S2 Text CONSORT statement. (PDF)
